# Heart Shaped Anterior Neck Lipoma Mimicking Thyroid Mass: A Case Report

**DOI:** 10.1002/ccr3.70098

**Published:** 2025-01-09

**Authors:** Abdus Samad Ansari, Dhiraj Chaurasia, Bikash Yadav, Season Shrestha, Inku Shrestha Basnet

**Affiliations:** ^1^ Kathmandu Medical College and Teaching Hospital Kathmandu Nepal; ^2^ Department of Otorhinolaryngology Kathmandu Medical College Teaching Hospital Kathmandu Nepal

**Keywords:** anterior neck lipoma, anterior neck swelling, case report, lipoma

## Abstract

Lipomas, one of the most common benign tumors, rarely occur in the anterior neck, often misdiagnosed as thyroid masses. This case highlights the diagnostic challenge posed by such lesions. A 58‐year‐old male presented with a painless slowly progressive anterior neck swelling initially mistaken for a thyroid mass. Though clinical examination findings suggested lipoma, the location of swelling influenced the decision toward thyroid mass. The elevated thyroid stimulating hormone (TSH) level further inclined the diagnosis toward thyroid mass. But imaging and fine needle aspiration cytology shifted the diagnosis toward a lipoma. Surgical excision was performed successfully. The gross and histopathological examination confirmed the diagnosis of lipoma. Anterior neck lipomas pose diagnostic challenges due to their uncommon location. Thorough clinical examination and evaluation is crucial to differentiate them from other neck pathologies, primarily thyroid mass. Surgical excision remains the preferred treatment. Postoperative follow‐up is important to monitor for recurrence and complications. Awareness of anterior neck lipoma is vital among clinicians for early diagnosis and optimal management. Early suspicion of this prevalent condition in the given unusual location can guide them towards appropriate diagnostic approach and treatment decisions, ultimately improving patient outcomes and reducing diagnostic complexity for healthcare professionals.

**Summary**

- Anterior neck lipomas are rare and often mistaken with more prevalent regional pathologies.
- Our case report presents a male with midline anterior neck swelling initially mistaken for thyroid mass; but proper clinical evaluation and investigation modalities confirmed the diagnosis as lipoma which was successfully excised surgically.

## 1 Introduction and Importance

Lipomas are one of the most frequently encountered entity in medical practice accounting for around 5% of all benign tumors [1]. While lipomas can originate in various regions of the body where adipose cells are present, only 13% manifest in the head and neck region. Within the neck, they predominantly surface in the posterior triangle, with lipomas in the anterior neck being rare [2]. Consequently, the lipoma as a diagnosis is frequently overlooked in clinical practice during initial diagnostic considerations of anterior neck swelling; often being mistaken with other prevalent neck pathologies, primarily thyroid mass. Here we present a case of 58 years old male with midline anterior neck lipoma posing a diagnostic challenge. This case report has been reported in accordance with the Surgical CAse REport (SCARE) guideline [3].

## 2 Case History/Examination

**FIGURE 1:**
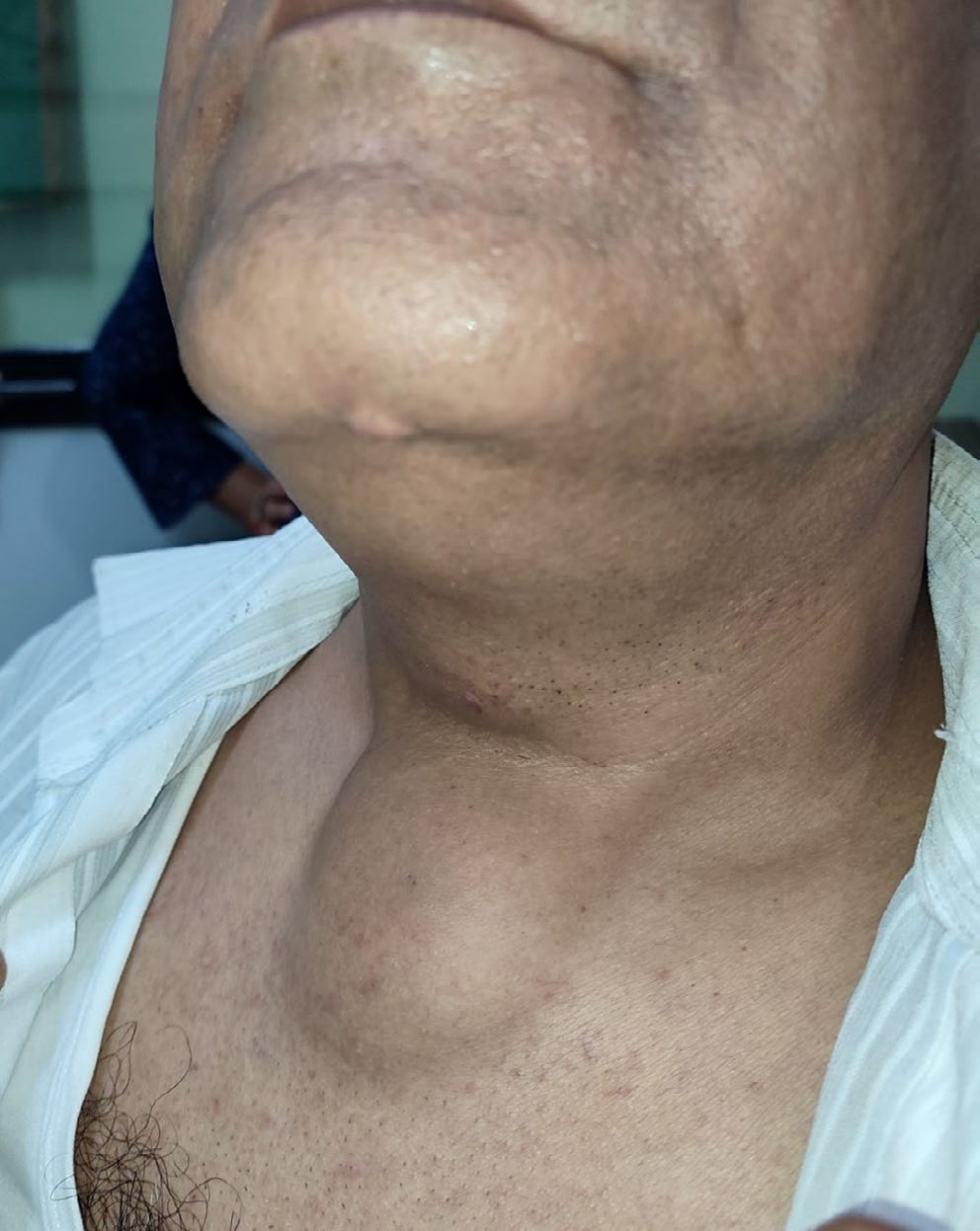
Anterior neck swelling at presentation.

A 58 years old male presented with slowly progressive but painless swelling over the midline of anterior neck initially noticed around 12 years ago, measuring around 6 cm × 5 cm at the time of presentation. The swelling (Figure 1) extended vertically from 6 cm below the ramus of mandible to 4 cm below the clavicle and horizontally 3 cm on each side from the midline. The swelling caused no symptom at all in the patient.

Clinical examination revealed globular, smooth, well defined, mobile and fluctuant swelling with regular margin and no overlying skin changes; however, there was neither local rise in temperature nor any presence of tenderness. The swelling was non‐compressible, non‐reducible, non‐pulsatile, and non‐trans illuminant. The swelling did not move with deglutition but elicited positive slipping sign. The patient did not have any palpable lymph node or audible bruit.

## 3 Methods

Given the patient's medical history and clinical examination, our differential diagnoses included lipoma and thyroid mass.

**FIGURE 2:**
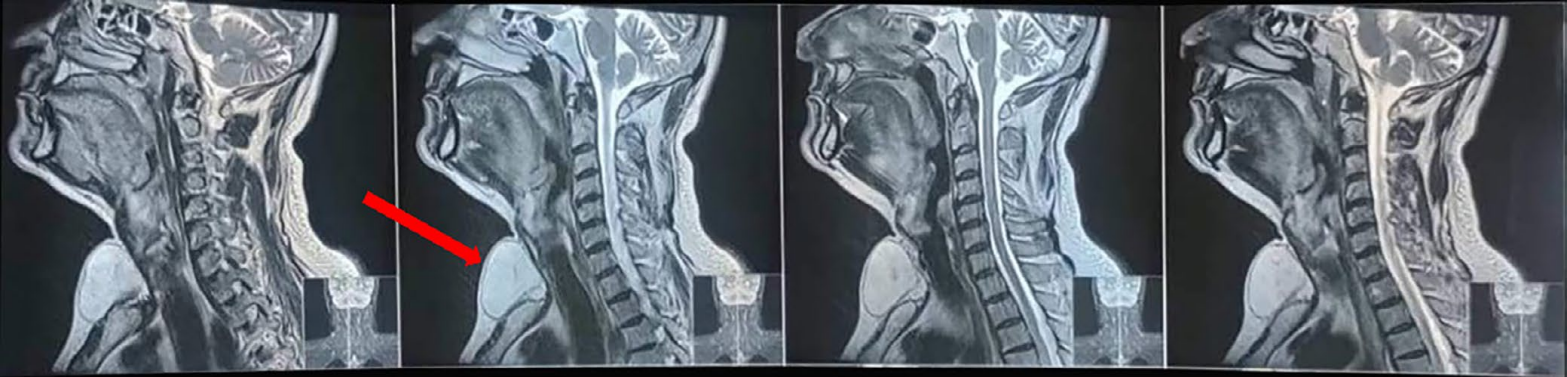
MRI (Sagittal section) neck image of the patient.

His thyroid stimulating hormone (TSH) level was slightly high (4.68 μIU/ml; Normal value: 0.3–4.5) tilting the scale of suspicion more toward a thyroid mass. However, ultrasonography (USG) findings suggested subcutaneous soft tissue lesion likely lipoma. Plain magnetic resonance imaging (MRI) of neck (Figure 2) demonstrated fat signal intensity mass in both T1 and T2 weighted imaging measuring approximately 6 × 6 × 5 cm in midline anterior neck. The mass was surrounded by a capsule with no retrosternal extension or pressure effects on surrounding tissues. Aforementioned features indicated characteristics consistent with lipoma. A fine needle aspiration was conducted for further certainty of the diagnosis, for which samples were collected from various levels of the swelling to ensure sufficient and representative cytological specimen. The cytopathological smears demonstrated fragments of mature adipose tissue with no nuclear atypia which was also suggestive of lipoma.

Based on the patient's medical history, clinical evaluation, and above mentioned investigative findings, a conclusive preoperative diagnosis of subcutaneous lipoma of anterior neck was established.

Patient underwent complete excision of the mass under general anesthesia through a nearly 5 cm transverse skin crease incision made approximately 4 cm below the thyroid cartilage. The mass was present in the subcutaneous plane and was not attached to any surrounding tissue. The presence of capsule around the mass significantly eased the process of tumor removal. The base was secured with ligature and subsequently transected.

**FIGURE 3:**
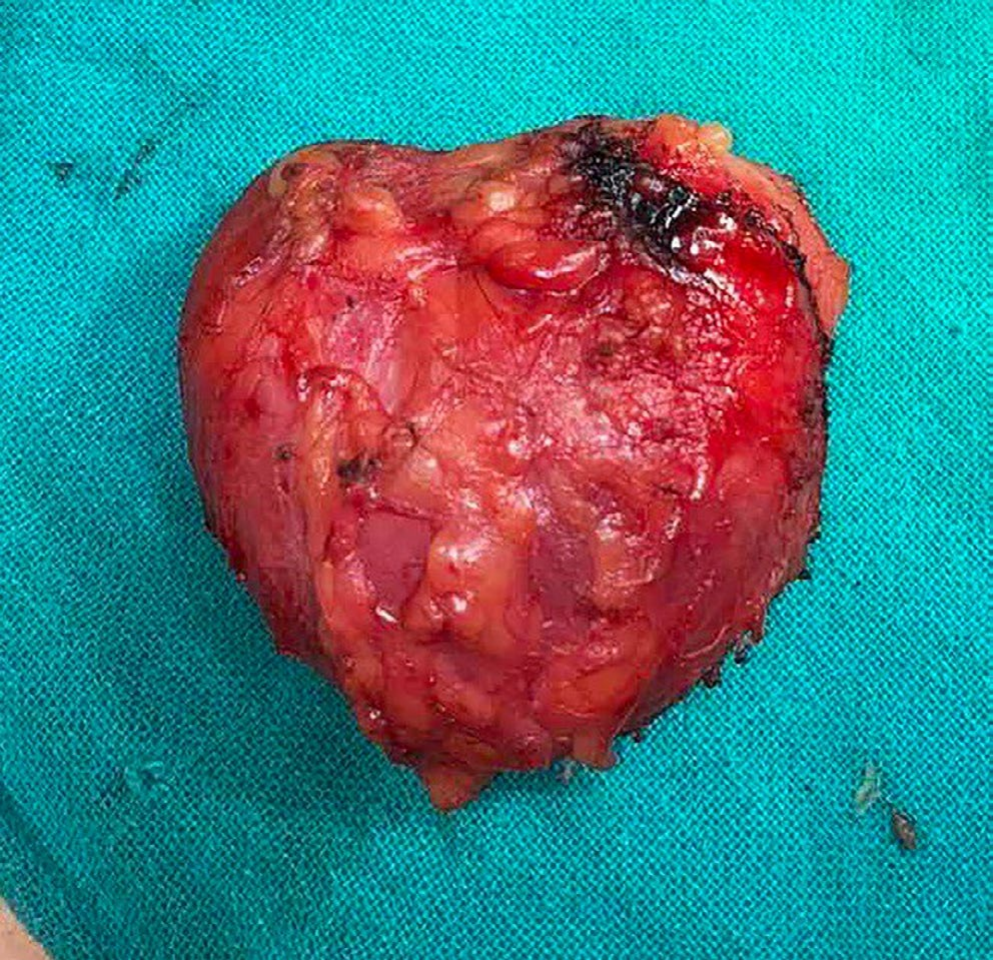
Gross specimen of lipoma.

**FIGURE 4:**
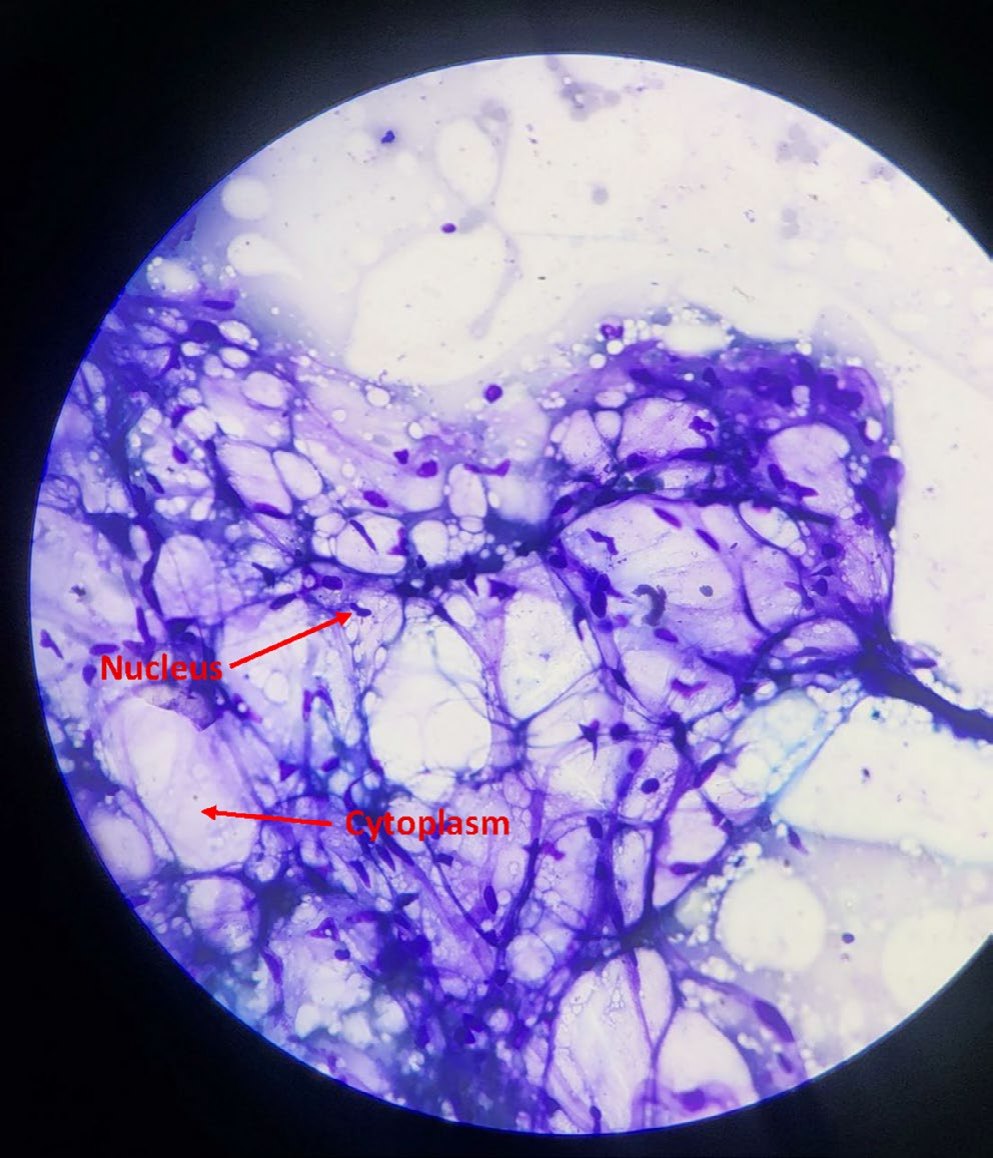
Histopathology specimen of lipoma (Giemsa staining; magnification 400X) showing adipocytes.

Grossly, the specimen (Figure 3) was heart shaped and yellowish in color and covered with thin capsule. It had dimension of around 8 × 9 cm with a weight of approximately 350 Grams. Cut surface revealed soft, glistening, yellowish greasy appearance. Histopathological examination (Figure 4) showed mature adipose tissue separated by fibrous septa with no malignant change which confirmed the diagnosis of lipoma.

## 4 Results

The postoperative period was uneventful allowing for the patient's discharge with a pleasing cosmetic results and no existing complications.

## 5 Discussion

Lipomas are benign mesenchymal tumors usually present in subcutaneous tissue originating from adipose cells [4]. They represent the most prevalent mesenchymal tumors, constituting approximately 50% of soft tissue neoplastic cases with an estimated annual occurrence of 1/1000 [5]. Despite their classification as universal or ubiquitous tumor, given their potential to develop in any region where fat cells are present [6, 7]; merely 13% of lipomas occur in the head and neck region [8], with the posterior triangle standing out as the most frequent location [1]. Lipomas involving the anterior neck region, however, are a rare occurrence [1, 9]. Although lipomas occur equally among males and females with no gender predominance and mostly present in fifth to sixth decade [10], lipomas in this particular area typically manifest in women during their fourth and fifth decades of life [11]. The patient under consideration in our case was a 58‐year old male.

Lipomas are characterized by their soft, mobile, non‐tender, and slow‐growing nature. They typically remain asymptomatic unless they compress surrounding organs or neurovascular structures [10]. The presence of these findings in our clinical assessments strongly indicated the presence of a lipoma; however, given its location, there was an equal inclination towards considering it as being a thyroid mass. Thus in anterior neck swelling, proper history and clinical examination with appropriate diagnostic tests are necessary to confirm the nature of the swelling and its association with surrounding regional organs. Other potential differentials for neck swelling, such as branchial cysts, thyroglossal cysts, hemangiomas and lymph nodes were considered. However, our initial clinical evaluation concluded these diagnoses as highly unlikely.

The size of the swelling in our patient was 8 × 9 cm which was not unusually large, but exceptionally large lipomas (largest measuring 32 cm in greatest dimension [12]) are also reported in previous literatures.

USG is the first diagnostic test recommended for any superficial swelling owing to its low cost and rapid results. The USG findings in this case were suggestive of soft tissue mass, likely lipoma. The possibility of a branchial cyst was also excluded due to the absence of posterior acoustic enhancement and the lack of a thin walled cystic lesion [13]. But considering the location of the mass, ruling out the relation of the swelling with surrounding organs and neurovascular structures required better imaging modality like MRI. MRI reveals a typical appearance for lipoma that is homogenous, hyperintense mass both on T1 and T2 weighted imaging with a signal similar to subcutaneous fat [14]. The MRI findings in this case demonstrated fat signal intensity mass surrounded by capsule with no retrosternal extension or pressure effects on surrounding tissues. Fine needle aspiration cytology (FNAC) is recommended in cases where the clinical diagnosis is uncertain or when the sonogram fails to clearly delineate the outline of lipoma [15]. FNAC was performed in this patient to provide an additional layer of assurance regarding the diagnosis and to exclude the presence of thyroid tissue; as rarely thyroid lesions are also found to contain adipose tissues [16]. On histopathology, lipomas are composed of mature adipose tissue.

The preferred treatment modality for most of the cases is surgical excision. In our patient, the horizontal skin crease incision along with the presence of capsule around the mass enabled us to effortlessly visualize and dissect the tumor, all the while ensuring preservation of adjacent neck organs and neurovascular structures. Utmost caution must be ensured throughout the dissection process, as the delicate integrity of the neck anatomy and neurovascular structures are susceptible to potential injury. Liposuction can also be an alternative to the excisional surgery [17]. Squeeze delivery through a small cosmetic incision has also demonstrated notable success in removal of lipomas located in the arms and legs. However, its success rate appears to be comparatively low when utilized for lipomas in the head and neck region [18]. In spite of clear evidence of advantages of both of these surgeries, the decision of going for traditional surgical method was influenced by the size of the mass and its close proximity to the major nerves and vessels in the neck. Steroid injections are another treatment option, particularly for small lipomas, however many injections may be required with the possibility of overlying skin pigmentation [19].

Considering Follow up is a sensible approach to monitor for any potential recurrence. Possible postoperative complications such as neurovascular injury, scar and asymmetric contour must be discussed with the patient both before the operation and upon discharge.

We compared our findings with similar cases documented in the literature. The accompanying table (Table 1) provides a summary of published reports on anterior neck lipomas, detailing patient demographics, chief complaints, lipoma locations, diagnostic modalities, and treatment approaches. This comparison highlights commonalities and differences in the presentation and management approaches, enhancing our understanding of the clinical nuances associated with anterior neck lipomas.

**TABLE 1:** Summary of published case reports on anterior neck lipomas.

Author	Published year	Age/sex	Location	Chief complain	Diagnostic modality used	Treatment	Outcome
Kabiri et al. [20]	2023	55/M	Left sided Lateral neck	Painless swelling	CT scan	Surgical excision	Uneventful postoperative period
Rashwan et al. [4]	2023	59/M	Anterior triangle of neck	Swelling with mild dysphagia	MRI	Surgical excision	—
Ongjen Cukic [21]	2020	37/F	Right sided anterior neck	Swelling with occasional dyspnea	MRI	Surgical excision	Uneventful postoperative period

## 6 Conclusion

Neck swellings are commonly encountered presentation, yet their potential differential diagnoses are diverse. Most of them are easily diagnosed but those at rare locations as in our case pose a difficulty in diagnosis. Recognizing a potential lipoma clinically can alter the diagnostic and treatment approach alleviating the complexity for healthcare professionals.

## Author Contributions

**Abdus Samad Ansari:** conceptualization, data curation, writing – original draft, writing – review and editing. **Dhiraj Chaurasia:** data curation, writing – original draft, writing – review and editing. **Bikash Yadav:** writing – original draft, writing – review and editing. **Season Shrestha:** conceptualization, data curation, supervision, validation. **Inku Shrestha Basnet:** conceptualization, supervision, validation.

## Consent

Written informed consent was obtained from the patient before the study for the publication of this Case Report.

## Conflicts of Interest

The authors declare no conflicts of interest.

## Data Availability

Data sharing not applicable ‐ no new data generated.
